# Rapid Classification and Identification of *Microcystis aeruginosa* Strains Using MALDI–TOF MS and Polygenetic Analysis

**DOI:** 10.1371/journal.pone.0156275

**Published:** 2016-05-26

**Authors:** Li-Wei Sun, Wen-Jing Jiang, Hiroaki Sato, Masanobu Kawachi, Xi-Wu Lu

**Affiliations:** 1 School of Energy & Environment, Southeast University, Nanjing, Jiangsu, China; 2 Environmental Measurement Technology Group, Environmental Management Research Institute, National Institute of Advanced Industrial Science and Technology, Tsukuba, Ibaraki, Japan; 3 Biodiversity Resource Conservation Section, Center for Environmental Biology and Ecosystem Studies, National Institute for Environmental Studies, Tsukuba, Ibaraki, Japan; University of New South Wales, AUSTRALIA

## Abstract

Matrix-assisted laser desorption–ionization-time-of-flight mass spectrometry (MALDI–TOF MS) was used to establish a rapid, simple, and accurate method to differentiate among strains of *Microcystis aeruginosa*, one of the most prevalent types of bloom-forming cyanobacteria. *M*. *aeruginosa* NIES-843, for which a complete genome has been sequenced, was used to characterize ribosomal proteins as biomarkers and to optimize conditions for observing ribosomal proteins as major peaks in a given mass spectrum. Thirty-one of 52 ribosomal subunit proteins were detected and identified along the mass spectrum. Fifty-five strains of *M*. *aeruginosa* from different habitats were analyzed using MALDI–TOF MS; among these samples, different ribosomal protein types were observed. A polygenetic analysis was performed using an unweighted pair-group method with arithmetic means and different ribosomal protein types to classify the strains into five major clades. Two clades primarily contained toxic strains, and the other three clades contained exclusively non-toxic strains. This is the first study to differentiate cyanobacterial strains using MALDI–TOF MS.

## Introduction

Toxic cyanobacterial blooms have been identified in fresh and brackish waterbodies for over 100 years [1]. They are known to cause the deaths of wild and domestic animals worldwide [2] and threaten the health of human beings who use waterbodies with such blooms for recreation or drinking [3].

*Microcystis aeruginosa* is one of the most predominant cyanobacterium found in freshwater bodies. Some strains produce hepatotoxins called microcystins (MCs), which have been suspected of causing human hepatocellular carcinoma in China [3]. The World Health Organization (WHO) has set the drinking water standard for MC-LR at 1 μg/L [4]. MCs exist within cyanobacteria and are released after cyanobacterial cells lyse. In addition, their distribution is highly correlated with the distribution of cyanobacteria. MC concentrations shift with the movement of cyanobacteria in the waterbodies where they occur, thus monitoring cyanobacteria indirectly allows researchers to monitor MCs.

Cell counting [5], pigment analysis [6, 7, 8], and 16S rRNA gene-based molecular techniques [9, 10] have been used to detect and monitor cyanobacteria in samples taken from naturally occurring sources. The disadvantages of these methods are that they are time-consuming and require a skilled expert to perform the analysis, thus limiting their application in routine monitoring. Therefore, more rapid, easy to use, and reliable monitoring methods are required.

Both toxic and non-toxic cyanobacterial strains coexist in the natural environment, and the uncertain distribution of both types of strains prevents the accurate diagnosis of blooms. As a result, the toxigenic potential of cyanobacterial populations has gone unidentified, and corresponding control measures have not been put in place.

The toxicity of cyanobacterial strains cannot be distinguished by microscope. Instead, several molecular typing methods have been established to distinguish toxic and non-toxic strains from one another. These methods include random amplified polymorphic DNA fingerprinting [11], 16S rDNA analysis [8, 10, 12], 16S–23S rDNA internal transcribed spacer analysis [13, 14], analysis of a segment of the phycocyanin operon cpcBA intergenic spacer [10, 11], and MC biosynthesis gene *mcy* analysis [10, 15, 16]. However, the results of these analyses have indicated that the toxicity of different strains does not always coincide with that strain’s gene type, even when the MC genes are employed as markers (i.e., strains of one *mcy* genotype range from toxic to non-toxic).

Rantala *et al*. [17] found that microcystin synthetase genes were present in the last common ancestor of a large number of cyanobacteria; however, the ability to produce the toxin has been lost in many of the more derived cyanobacteria lineages. Therefore, the toxicity of any given strain must be determined by a method with a finer resolution than the microcystin synthetase gene level.

Tanabe *et al*. [18] developed the multilocus sequence typing (MLST) method based on seven selected housekeeping loci that could distinguish toxic from the non-toxic strains at the genetic level. However, their method is extremely complex, labor intensive, and time-consuming.

In this study, we propose a new approach to this process by using matrix-assisted laser desorption ionization (MALDI) time-of-flight (TOF) mass spectrometry (MS) to create a rapid, high resolution method for identifying and distinguishing toxic *M*. *aeruginosa* strains from non-toxic strains.

Several reports have demonstrated the feasibility of using MALDI-TOF-MS to identify microorganisms [19–26]. In whole-cell MALDI–TOF MS, characteristic “fingerprint” spectra are obtained from whole (intact) cells, eliminating the biomarker pre-fractionation, digestion, separation, and cleanup steps. The procedure is quick and requires a minimal amount of biological material (sub-colony amounts); therefore, it is suitable for high-throughput routine analysis and has great potential for application in clinical microbiology and environmental monitoring.

The protein biomarkers observed in the mass spectrum are typically highly expressed proteins with housekeeping functions, such as ribosomal or nucleic acid-binding proteins [19, 27, 28]. These proteins are highly conserved in bacteria, which means they may have universal application. Pineda *et al*. [27] were first to propose the use of ribosomal proteins as biomarkers in the identification of bacteria using MALDI–TOF MS; and in our previous studies, we characterized 42 ribosomal proteins from a typical lactic acid bacterium, *Lactobacillus plantarum*, using both two-dimensional gel electrophoresis confirmation and MALDI–MS determination [29]. The ribosomal proteins within *L*. *delbrueckii* subsp. *bulgaricus* and *Streptococcus thermophiles* have also been characterized through a comparison of MALDI–TOF MS observation data and available public protein sequence databases [30]; furthermore, the observed masses of some ribosomal subunit proteins have been found to vary within the species, indicating the possibility of classifying bacteria at the strain level. Finally, polygenetic classification of *Pseudomonas putida* strains has occurred using MALDI–TOF MS with ribosomal proteins as biomarkers [31].

In this study, an optimized pre-treatment method to observe ribosomal proteins as major peaks in the mass spectrum and to characterize ribosomal proteins as biomarkers was tested. Fifty-five strains of *M*. *aeruginosa* from different habitats were then assembled, and their different ribosomal protein types were analyzed using MALDI–TOF MS. A polygenetic analysis using the ribosomal proteins from the 55 *M*. *aeruginosa* strains revealed high genetic diversity among the strains. Finally, these strains were further assembled into five major clades based on toxicity.

## Materials and Methods

### *M*. *aeruginosa* strains

Information about the 55 *M*. *aeruginosa* strains used in this study is listed in S1 Table. The strains were provided by the Microbial Culture Collection at the National Institute for Environmental Studies (MCC-NIES, Tsukuba, Japan). Most of the strains were isolated from freshwater environments in Japan, but the group also included strains (one each) from each of the following countries: Germany, the United Kingdom, Thailand, and Nepal. All but one of the strains (NIES-478) was a clone. Cultures were grown in an MA medium [32] at 20°C for 4 weeks under a 12:12-h light:dark cycle with a photon density of 15 μmol m^−2^ s^−1^. Of the sample strains, the genome-sequenced strain NIES-843 was used to characterize the ribosomal proteins and optimize the sample preparation method.

### Preparation of intact cells

First, because *M*. *aeruginosa* cells have gas vesicles for buoyancy, a mild sonication treatment was applied. The cells were centrifuged at 9,100 *g* for 10 min in a 15-mL tube, and then the precipitate was transferred to a 1.5-mL tube and centrifuged at 20,400 *g* for 10 min. The resulting pellet was re-suspended with 10 μL of a 50% acetonitrile containing 1% trifluoroacetic acid solution and analyzed using MALDI–TOF MS.

### Preparation of cell lysates

The cell pellet (wet weight ranged from 20–40 mg and was measured during the last preparation step) was suspended in a 160 μL of TMA-1 buffer [10 mM Tris-HCl (pH 7.8), 30 mM NH_4_Cl, 10 mM MgCl_2_, and 6 mM 2-mercaptoethanol], transferred to a tube containing 160 mg zirconia silica beads, and ground at 3,000 rpm with a Mini Bead-Beater (Biospec products) for 1 min. The beads and cell debris were removed by centrifuging the mixture at 5,800 *g* for 25 min, and a portion of the resulting cell lysate was analyzed using MALDI–TOF MS.

### Preparation of the concentrated ribosomal fraction

The cell lysate was adjusted to 250 μL with TMA-1 buffer and then centrifuged using an Allegra 64R (Beckman Coulter, Brea, CA, USA) at ca. 64,000 g for 4 hours. The concentrated ribosomal fraction was collected in the precipitate. The precipitate was re-suspended with 10 μL of 50% acetonitrile containing 1% trifluoroacetic acid solution and analyzed using MALDI–TOF MS.

### MALDI–TOF MS

The sample preparation, apparatus, and MALDI–TOF MS data acquisition techniques used in this study were similar to those described in our previous papers [29, 30]. Each sample solution was mixed with a 20 mg mL^−1^ of sinapinic acid matrix solution in 50% acetonitrile containing 1% trifluoroacetic acid. Approximately 2 μL of the sample matrix mixture was placed onto the MALDI target and dried in open air. All MALDI mass spectra in the range *m/z* 2000–40000 were recorded using the positive linear mode by averaging 500 individual laser shots from an AXIMA CFR plus time-of-flight mass spectrometer (Shimadzu/Kratos, Kyoto, Japan) equipped with a pulsed N_2_ laser (λ, 337 nm; pulse width, 3 ns; frequency, 10 Hz).

### Cluster Analysis

The mass-matching profiles for each sample strain were processed to build a dendrogram using the unweighted pair group method with arithmetic mean (UPGMA) cluster analysis dendrogram and a categorical coefficient. Analyses were conducted using BioNumerics software, version 3.5 (Applied Maths, Kortrijk, Belgium).

## Results

### Characterization of the ribosomal subunit proteins of *M*. *aeruginosa* NIES-843

First, a list of the calculated molecular weights corresponding to the ribosomal subunit proteins of the *M*. *aeruginosa* strain NIES-843 was prepared (Table 1). The complete NIES-843 genome had already been sequenced [33]. The ribosomal protein amino acid sequences were retrieved from a public protein database (UniProtKB: http://www.uniprot.org/), and then the theoretical molecular weight of each ribosomal protein was calculated using the Compute pI/MW tool (http://www.expasy.org/compute_pi/). The most common post-translation modification rule “N-end rules” [34] was used to determine the possible protonated versions of each protein in the form [M+H]^+^ (Table 1).

**Table 1:** Assigned ribosomal subunit proteins of *M*. *aeruginosa* NIES 843.

protein name	mass of [M+H]	post-translational modification	second amino acid residue	UniProtKB/Swiss-Prot: access number
**Small Subunit Proteins**				
S08	14557.9	-Met	S	B0JHZ0.1
S09	15017.3		Q	B0JY34.1
S13	14290.4	-Met	A	B0JY40.1
S14	11836.6	-Met	A	B0JYC2.1
S15	10245.7	-Met	A	B0JPD9.1
S16	9561.1		I	B3DFB2.1
S17	8998.4	-Met	A	B0JHZ4.1
S18	8204.6	-Met	S	B0JVN4.1
S19	10128.8	-Met	G	B0JHZ9.1
S20	10420.1	-Met	A	B0JGK4.1
S21	7094.3	-Met	T	B0JIQ1.1
**Large Subunit Proteins**				
L06	19941.1	-Met	S	B0JHY9.1
L09	16662.3	-Met	A	B0JQ94.1
L13	16923.7		N	B0JY35.1
L14	13305.5		I	B0JHZ3.1
L15	15831.4		K	B0JHY6.1
L17	12964.2		R	B0JY37.1
L18	13250.1		K	B0JHY8.1
L19	13847.3		K	B0JNQ1.1
L21	14516.4	-Met	S	B0JI00.1
L22	13232.4	-Met	T	B0JHZ8.1
L23	11257.3	-Met	V	B0JI01.1
L24	12840.9	-Met	S	B0JHZ2.1
L28	8971.5	-Met	S	B0JLJ8.1
L29	8231.6	-Met	A	B0JHZ5.1
L31	8751.1	-Met	P	B0JY33.1
L32	6719.5	-Met	A	B3DFA5.1
L33	7270.4	-Met	A	B0JVN3.1
L34	5235.2	-Met	S	B0JN63.1
L35	7866.4	-Met	P	B0JSJ9.1
L36	4399.4		K	B3DFA8.1

**Fig 1:**
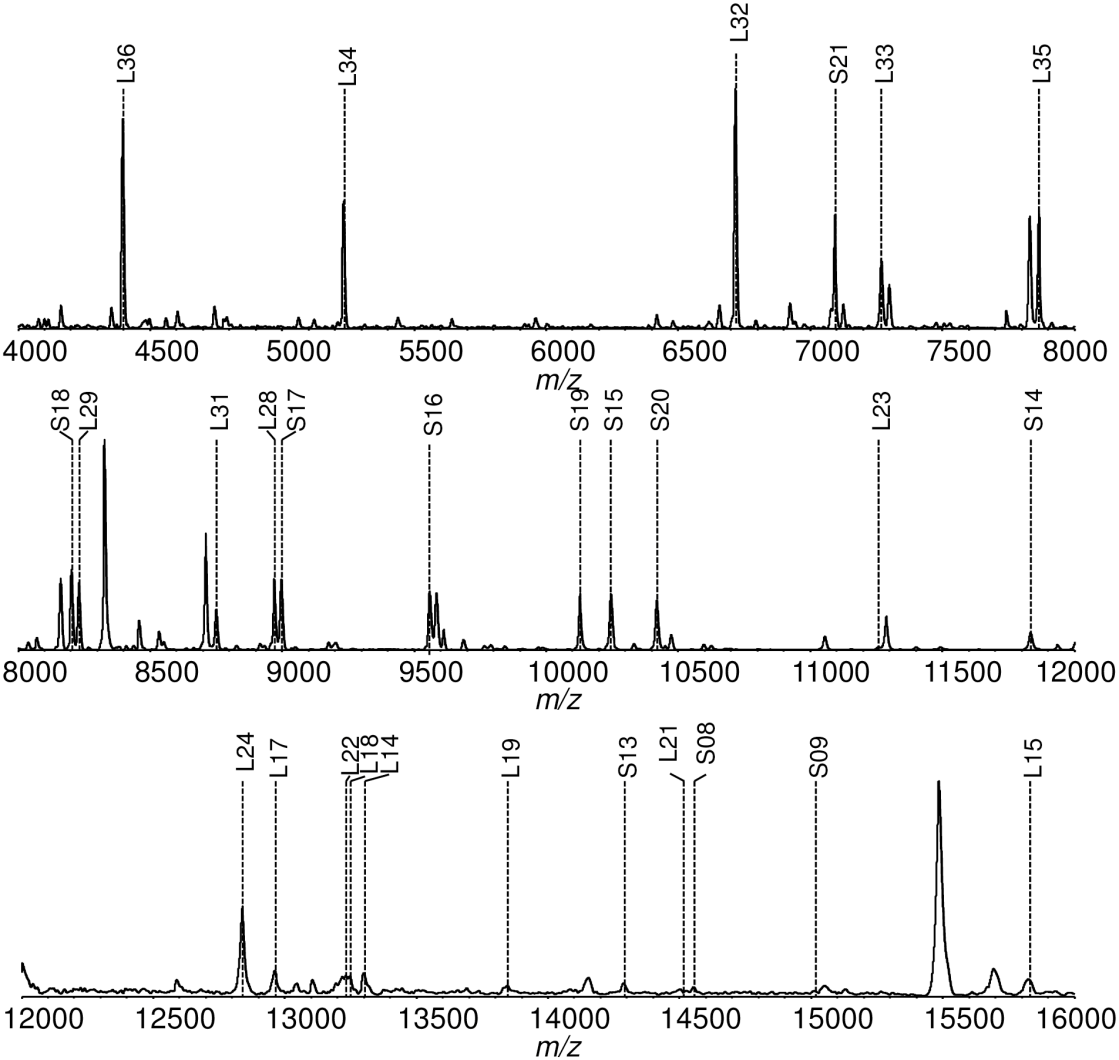
Mass spectra of the *M*. *aeruginosa* (NIES-843) ribosomal proteins and their peak assignments. Peaks that are identified as ribosomal proteins are marked with the protein’s name.

Fig 1 shows the mass spectrum of the NIES-843 strain from *m/z* 4000–16000. The *m/z* values of the detected peaks were compared to the calculated masses [M+H]^+^ of the ribosomal proteins. Thirty-one of the 52 ribosomal proteins were identified in the mass spectrum. Those located between *m/z* 4000–16000 are labeled in Fig 1. Table 1 summarizes all the assigned ribosomal proteins from *M*. *aeruginosa* NIES-843, along with their calculated masses and possible post-translation modifications.

### Comparison of the samples’ mass spectra values using three different pre-treatment methods

**Fig 2:**
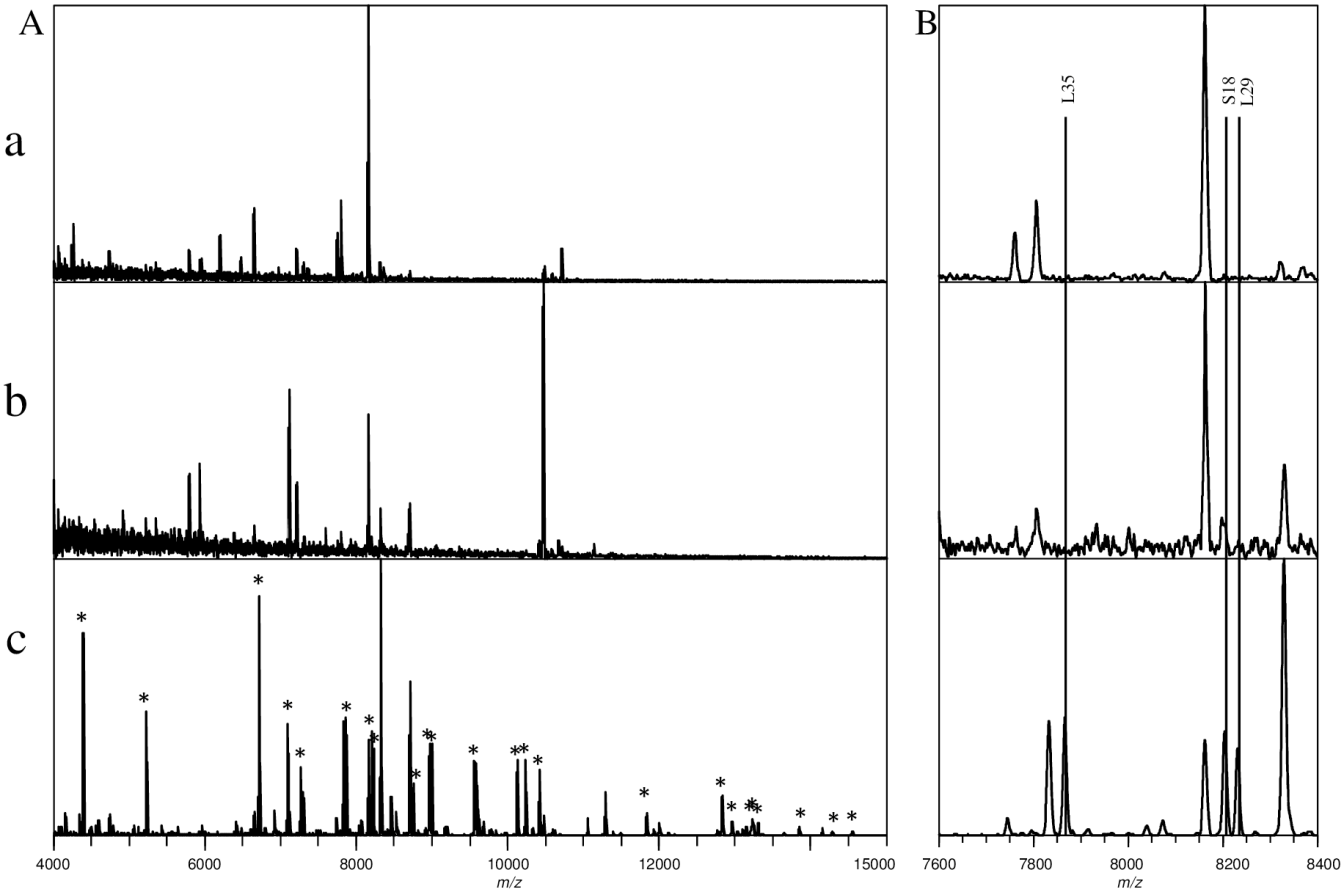
Comparison of the spectra of *M*. *aeruginosa* (NIES-843) samples prepared using different pre-treatment methods. a: intact cell; b: cell lysate; c: concentrated ribosomal proteins. A: mass spectra (*m/z* 4000–15000) of three pre-treatment methods. Peaks marked by * are ribosomal proteins identified from concentrated ribosomal sample; B: Expanded view of the three pre-treatment methods’ mass spectra (*m/z* 7600–8400), in which ribosomal proteins L35, S18 and L29 were identified from the concentrated ribosomal sample.

Fig 2 illustrates the mass spectra of samples prepared using three different pre-treatment methods. For the intact cell mass spectrum, many peaks could be observed; but these peaks could not be attributed to ribosomal proteins (Fig 2a). For the cell lysate pre-treatment, the lysate solution was green and slightly viscous, which is primarily attributable to the pigment (chlorophyll) and a mucilage composed of polysaccharides. As a result, the observed peak intensities of the mass spectrum for this pre-treatment exhibited a reduced signal-to-noise ratio, and no ribosomal proteins were observed (Fig 2b). Because ribosomal proteins should exist in the cell lysates, high-speed centrifugation was performed to collect the ribosomal fraction of the cell lysate. The mass spectrum observed for the collected ribosome fraction showed clear ribosomal protein peaks, as seen in Fig 2c. Thirty-one ribosomal proteins were identified in this mass spectrum (Table 1).

### Polygenetic analysis of *M*. *aeruginosa* strains

At present, the genomes of 12 *M*. *aeruginosa* strains have been completely or partially sequenced. As shown in Table 2, the molecular weights of some of the ribosomal proteins identified varied among different strains because the amino acid sequences of these ribosomal proteins differed. By identifying the ribosomal proteins from *M*. *aeruginosa* NIES-843 as Type I, other types of ribosomal proteins could be identified and placed in numerical order. Several types of ribosomal proteins have been identified among these different strains.

**Table 2:** Ribosomal protein list for 12 sequenced strains.

Ribosomal proteins	MW (mass of [M+H]	NIES-843	PCC 7941	PCC 9432	PCC 9443	PCC 9701	PCC 9806	PCC 9808	THAIHU98	DIANCHI905	PCC 9717	PCC 9807	PCC 9809
L32	6719.5	I (6719.5)	I (6719.5)	-	-	II (6647.39)	I (6719.5)	-	-	-	-	-	-
S21	7094.3	I (7094.3)	I (7094.3)	I (7094.3)	I (7094.3)	I (7094.3)	I (7094.3)	I (7094.3)	-	-	-	-	-
L33	7270.4	I (7270.4)	I (7270.4)	I (7270.4)	I (7270.4)	I (7270.4)	I (7270.4)	-	I (7270.4)	-	-	-	-
L35	7866.4	I (7866.4)	II (7844.29)	III (7843.31)	III (7843.31)	I (7866.4)	I (7866.4)	III (7843.31)	-	-	-	I (7866.4)	-
S18	8204.6	I (8204.6)	I (8204.6)	I (8204.6)	I (8204.6)	I (8204.6)	I (8204.6)	I (8204.6)	-	I (8204.6)	-	-	-
L29	8231.6	I (8231.6)	II (8291.56)	II (8291.56)	III (8318.62)	IV (8305.58)	III (8318.62)	II (8291.56)	VII (8247.55)	II (8291.56)	V (8290.57)	III (8318.62)	VI (8261.57)
L31	8751.1	I (8751.1)	II (8737.07)	II (8737.07)	II (8737.07)	III (8765.13)	I (8751.1)	II (8737.07)	-	-	-	-	-
L28	8971.5	I (8971.5)	II (8943.47)	I (8971.5)	III (8957.46)	I (8971.5)	I (8971.5)	I (8971.5)	-	-	-	-	-
S16	9561.1	I (9561.1)	I (9561.1)	I (9561.1)	I (9561.1)	II (9531.04)	II (9531.04)	I (9561.1)	-	-	-	-	-
S19	10128.8	I (10128.8)	I (10128.8)	I (10128.8)	I (10128.8)	I (10128.8)	I (10128.8)	I (10128.8)	-	-	-	-	-
S15	10245.7	I (10245.7)	II (10261.73)	II (10261.73)	II (10261.73)	I (10245.7)	III (10275.76)	II (10261.73)	I (10245.7)	II (10261.73)	I (10245.7)	II (10261.73)	I (10245.7)
S20	10420.1	I (10420.1)	I (10420.1)	II (10436.06)	III (10539.14)	IV (10367.02)	V (10450.09)	VI (10422.03)	-	-	-	-	-
S14	11836.6	I (11836.6)	II (11806.54)	II (11806.54)	II (11806.54)	II (11806.54)	II (11806.54)	II (11806.54)	-	-	-	-	-

Thirteen of the 31 ribosomal proteins in the NIES-843 strain (L32, S21, L33, L35, S18, L29, L31, L28, S16, S19, S15, S20, S14)—those that were frequently detected in all 55 strains and appeared at high intensities between *m/z* 4000–12000—were selected as biomarkers and classified by type, as summarized in Table 2.

**Fig 3:**
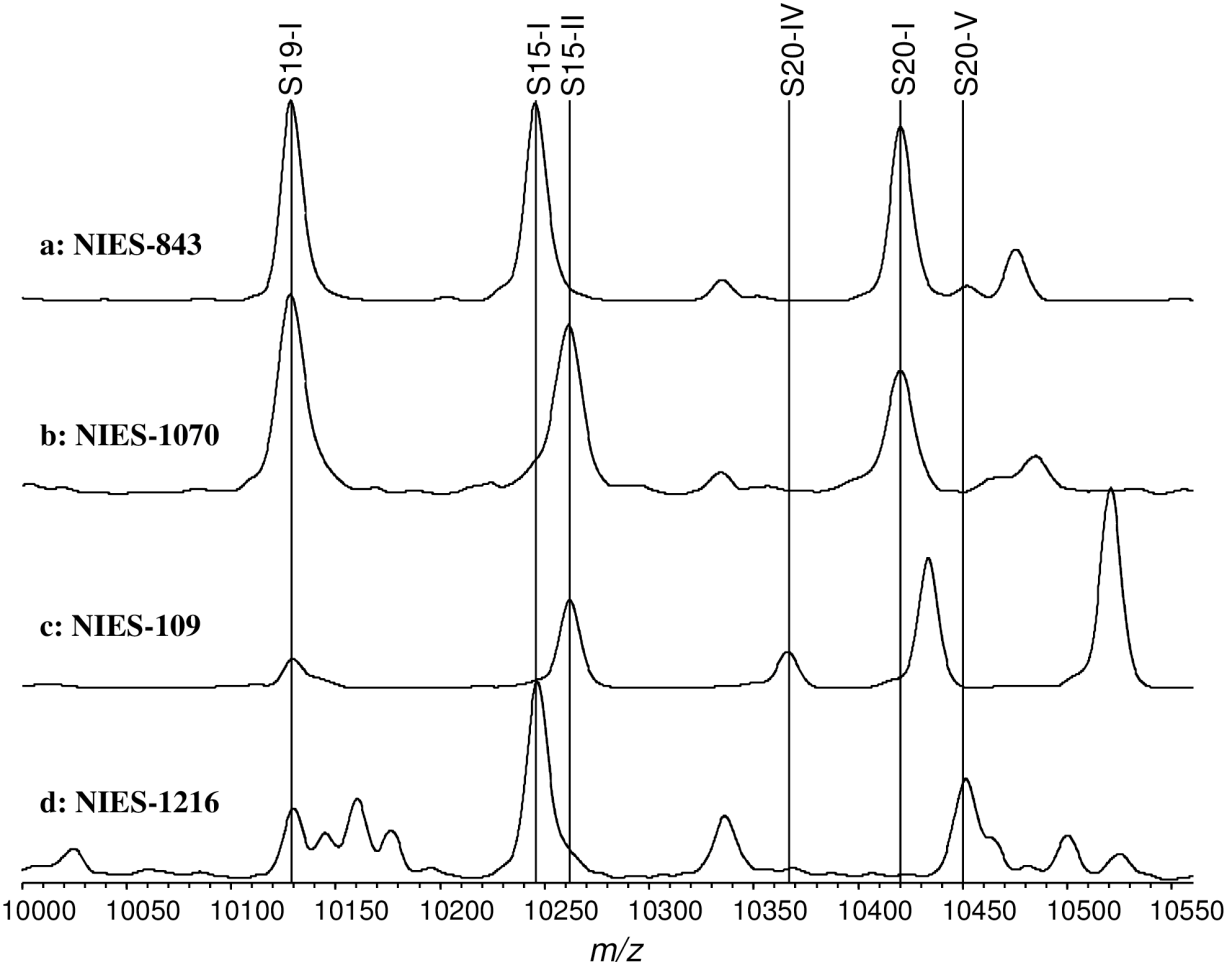
Expanded view of ribosomal proteins S19, S15, and S20 (*m/z* 10000–10500) from four strains. a: NIES 843; b: NIES 1070; c: NIES 109; d: NIES 1216. S19, S15, and S20 types are indicated by name.

Fig 3 shows the expanded mass spectra (*m/z* 10000–10550) of 4 *M*. *aeruginosa* strains (NIES-834, NIES-1070, NIES-109, and NIES-1216) as an example. These strains were selected from groups A, B, C, and D, respectively, as reported by Tanabe using MLST [18]. Ribosomal proteins S19, S15, and S20 are located in this mass spectrum range. Different versions of these three ribosomal proteins were identified in the mass spectra of the four example strains noted above. S19 exhibited the same mass (Type I) in all four strains. There were two forms of S15 among the four strains (Type I in NIES-843 and NIES-1216, and Type II in NIES-1070 and NIES-109). S20 exhibited more diversity among the four strains (Type I in NIES-843 and NIES-1070, Type IV in NIES-109, and Type V in NIES-1216). These results reveal that the four strains selected can be classified into different categories using the differences in their ribosomal proteins (Table 3).

**Table 3:** Different S19, S15 and S20 ribosomal protein types for four strains.

Strain	Ribosomal protein types		
	S19	S15	S20
NIES-843	1	1	1
NIES-1070	1	2	1
NIES-109	1	2	4
NIES-1216	1	2	5

**Fig 4:**
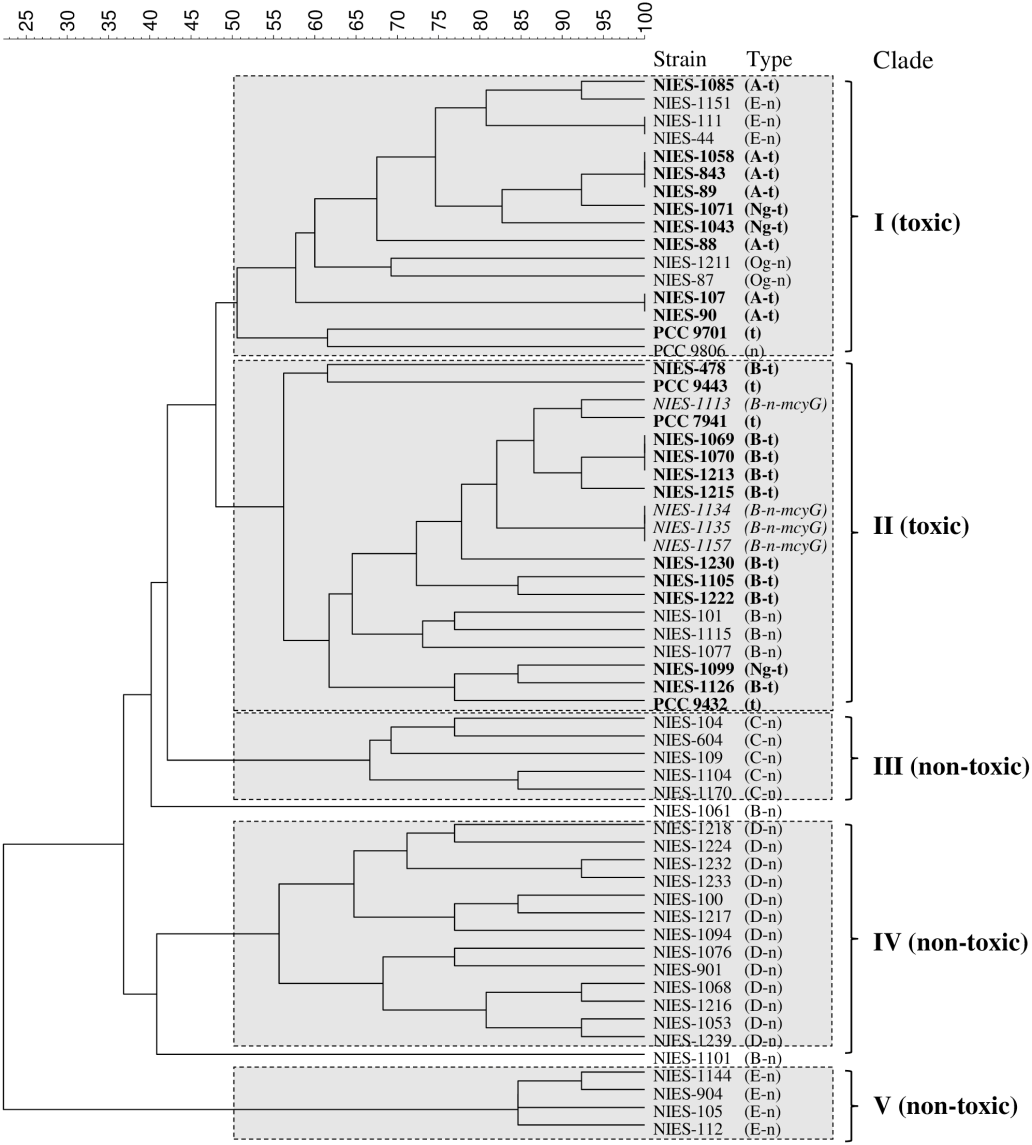
Polygenetic tree of *M*. *aeruginosa* strains based on MAIDI-TOF MS ribosomal protein profiling. Strains are labeled and classified by their group and toxicity. The group names indicated in capital letters (A, B, C, D, E) were described by Tanabe (2007) using the MLST method. t: toxic; n: non-toxic; Ng-no group; Og: other group. In addition to the measured strains, 5 sequenced strains (PCC 7941, PCC9432, PCC 9443, PCC 9701, PCC 9806) were employed as references.

All 55 strains were classified following the above pattern using the different types identified for the 13 selected biomarkers, and a ribosomal protein-profiling table (S2 Table) was prepared. The ribosomal protein-profiling table was then processed using UPGMA cluster analysis, and a polygenetic tree was created, as shown in Fig 4.

Based on the differentiation of the 13 biomarkers by type, the 55 strains were categorized into 47 unique strain types, highlighting the high diversity of the *M*. *aeruginosa* strains. These strain types were divided into five major clades (**I**–**V**) using a similarity cut-off of 50%. Notably, Clades **I** and **II** were dominated by strains that possessed *mcy*G, although several exceptions were seen. Clades **III**, **IV**, and **V** included strains that did not possess *mcy*G. Thus, the toxic strains (which possessed *mcy*G, labeled in bold by strain name and indicated by a t in Fig 4) and non-toxic strains (which did not possess *mcy*G, indicated with an n in Fig 4) were divided into different groups based on their ribosomal protein types, as determined using MALDI–TOF MS. These results are very similar to the results of an MLST analysis previously used to distinguish toxic and non-toxic strains of cyanobacteria [18].

## Discussion

In this study, 31 of the 52 ribosomal proteins from *M*. *aeruginosa* NIES-843 were successfully identified using MALDI–TOF MS, indicating that by employing ribosomal proteins as biomarkers, cyanobacteria can be identified using MALDI–TOF MS.

It has been reported that ribosomal proteins can be detected directly from whole-cell samples of Gram-negative bacteria [35]. Although *M*. *aeruginosa* is a Gram-negative bacterium, it has a special cell wall and extracellular covering that includes a relatively thick peptidoglycan layer sandwiched between outer and inner membranes, as well as a coating of polysaccharide fibrils external to the outer membrane [36]. This complex cell wall structure may prevent the release of cellular contents and impede the effective ionization of ribosomal protein fractions.

The successful detection and identification of ribosomal proteins from the concentrated ribosome fraction demonstrates that a two-step pre-treatment procedure is an effective method to prepare *M*. *aeruginosa* samples for ribosomal protein identification. Because the process included only cell lysis and centrifugation, it can be considered a simple, rapid study method.

Thus far, identification methods related to *M*. *aeruginosa* could only be used to identify the genus and species, even when 16S rRNA gene analysis was employed. The present results suggest that combining high resolution MALDI–TOF MS and using ribosomal proteins as biomarkers provides an accurate method to distinguish multiple strains of *M*. *aeruginosa*. Further, these results prove that differences in *M*. *aeruginosa* can be distinguished at strain level.

In the primarily toxic Clade **II** (Fig 4), four strains (NIES-1113, NIES-1134, NIES-1135, and NIES-1157) that possessed *mcy*G but did not produce MCs were identified. According to Rantala *et al*. [17], MC synthetase genes were originally present in all toxic strains of *Microcystis*, but some of these strains have lost the ability to produce MCs. Thus, the lack of toxicity in the four strains included in Clade **II** may stem from the fact that these strains lost their ability to produce MCs during evolution. The fact that these four strains fell into a toxic group and not a non-toxic group indicates the high discrimination ability of the MALDI-MS method and emphasizes the consistency between the results found in this study using ribosomal protein profiling and those found using *mcy*G gene-level analysis. Furthermore, three of these strains formed a monophyletic sub-clade, and the last strain was located quite near this clade. This may indicate that *mcy*G retained in these strains acts differently compared to other MC-producing genes.

Several non-toxic strains that did not possess *myc*G (NIES-1151, NIES-111, NIES-44, NIES-1211, NIES-87, NIES-101, NIES-1115, and NIES-1077) were also grouped in the otherwise toxic Clades (**I** and **II)**. Rantala *et al*. [17] have suggested that the phylogenetic marker 16S rRNA gene and the microcystin synthetase gene are congruent; as a result, the fact that these strains do not possess the *myc*G gene but still fall into a toxic clade may imply a relative relationship to those strains that possess *myc*G.

Without exception, all three non-toxic clades (Clade **III**-**V**) exclusively contained strains that do not possess the *mcy*G gene. This further demonstrates the high level of consistency between ribosomal protein profiling results and the MCs gene-typing process results. These results are significant because they demonstrate that toxic strains are not improperly identified when the method is applied to distinguish toxic and non-toxic strains.

The profiling of ribosomal proteins directly divided toxic and non-toxic *M*. *aeruginosa* strains into different clades. This suggests that measuring the ribosomal proteins of unknown strains or samples from natural waterbodies can be used to locate those strains within a toxic or non-toxic clade and provides a novel method to distinguish toxic *M*. *aeruginosa* strains within natural cyanobacterial samples. By continuing to analyze the mass spectrum database of *M*. *aeruginosa* strains, the accuracy of this method can be further improved.

Differentiating between toxic and non-toxic cyanobacteria strains is important to environmental management professionals because it can provide guidance on how to control these strains. Furthermore, the detection of toxic *M*. *aeruginosa* strains can help predict the release of MCs and allow for evaluations of the ecological risk of *M*. *aeruginosa* blooms in eutrophic waterbodies.

The polygenetic diversity of *M*. *aeruginosa* strains based on their ribosomal proteins is consistent with the MLST results presented by Tanabe [18] at the genetic level, which illustrates that ribosomal proteins are powerful markers in the study of polygenetic diversity. Theoretically, there are 52 ribosomal proteins in *M*. *aeruginosa* encoded by 52 genes. These proteins may provide additional genetic information beyond the seven housekeeping loci presented in Tanabe’s study. The study of these proteins only requires a two-step pre-treatment and MS measurement, which is much less time- and labor-intensive than previous strain analysis methods.

To our knowledge, the use of MALDI–TOF MS to analyze ribosomal proteins as biomarkers and distinguish toxic from non-toxic *M*. *aeruginosa* strains is a new concept and technique in the study of cyanobacteria. The simple pre-treatment and measurement process presented here enables the analysis of numerous samples in a short time period, such as in multi-point monitoring along or around a bloom’s breakout site. Furthermore, because MALDI–TOF MS is an analytical chemistry method, the process can be automated and standardized. Thus, it has the potential to be applied in routine monitoring of cyanobacterial water blooms.

For a given species of cyanobacteria, the potential toxicity of a proliferating population varies greatly not only depending on the location of a bloom but also how long that bloom exists [37, 38]. Although no such data are available in most eutrophic waterbodies, the MALDI–TOF MS method presented here may allow for an analysis of spatiotemporal changes in the toxicity of cyanobacterial blooms, particularly given its ability to rapidly and accurately predict toxicity.

Future studies may address the parameters linking the characteristics of a cyanobacterial bloom, including local biological, physicochemical, and weather conditions, to that bloom’s strain type. Understanding these conditions may provide insight into the mechanism of water bloom formation and subsequent control techniques. We plan to apply the results of this study to distinguish toxic strain types in natural samples and compare strain types from different eutrophic lakes, such as Kasumigaura Lake in Japan and Lake Taihu in China, in order to reveal the diversity of *M*. *aeruginosa* strains in different geographical regions.

## Supporting Information

S1 Table *M*. *aeruginosa* strain information. (PDF)

S2 Table Ribosomal protein peak typing of 55 strains. (PDF)
