# Correction: Rapid Classification and Identification of *Microcystis aeruginosa* Strains Using MALDI-TOF MS and Polygenetic Analysis

**DOI:** 10.1371/journal.pone.0170637

**Published:** 2017-01-18

**Authors:** The PLOS ONE Staff

The first two authors, Li-Wei Sun and Wen-Jing Jiang, should be noted as contributing equally to this work. The publisher apologizes for the error.
