# Supplementary material for: Rapid Classification and Identification of Microcystis aeruginosa Strains Using MALDI–TOF MS and Polygenetic Analysis
Source: PLoS One. 2016 May 26;11(5):e0156275. doi: 10.1371/journal.pone.0156275 (PMC4881969; doi:10.1371/journal.pone.0156275)
Supplement: S1 Table — (PDF) [file pone.0156275.s001.pdf]

Table S1 *M. aeruginosa* stains information

Strain number	Locality	Year	Remarks
NIES-44	Japan, Lake Kasumigaura	1974	Cryopreserved
NIES-87	Japan, Lake Kasumigaura	1982	Cryopreserved
NIES-88	Japan, Lake Kawaguchi	1981	Cryopreserved
NIES-89	Japan, Lake Kawaguchi	1981	Cryopreserved
NIES-90	Japan, Lake Kawaguchi	1981	Cryopreserved
NIES-100	Japan, Lake Suwa	1982	Cryopreserved
NIES-101	Japan, Lake Suwa	1982	Cryopreserved
NIES-104	Japan, Imperial Palace	1982	Cryopreserved
NIES-105	Japan, Lake Kasumigaura	1982	Cryopreserved
NIES-107	Japan, Lake Kawaguchi	1981	Cryopreserved
NIES-109	Japan, Lake Yogo	1982	Cryopreserved
NIES-111	Japan, Lake Kasumigaura	1978	Cryopreserved
NIES-112	Japan, Lake Suwa	1982	Cryopreserved
NIES-478	Japan, Lake Kasumigaura	1997	Cryopreserved
NIES-604	Japan, Lake Kasumigaura	1977	Cryopreserved
NIES-843	Japan, Lake Kasumigaura	1997	Cryopreserved
NIES-901	U.K. Scotland Dundee	1997	Cryopreserved
NIES-904	Thailand	1996	-
NIES-1043	Germany, Brandenburg Neuglobsow	2000	Cryopreserved
NIES-1053	Japan, Lake Kasumigaura	1978	Cryopreserved
NIES-1058	Japan, Lake Kasumigaura	1978	Cryopreserved
NIES-1061	Japan, Lake Suwa	1982	Cryopreserved
NIES-1068	Japan,Rokusuke-ike Pond	1982	-
NIES-1069	Japan, Rokusuke-ike Pond	1982	Cryopreserved
NIES-1070	Japan, Rokusuke-ike Pond	1982	Cryopreserved
NIES-1071	Japan, Ichinomiya-machi	1982	Cryopreserved
NIES-1076	Japan, Lake Yogo	1984	Cryopreserved
NIES-1077	Japan, Lake Yogo	1984	Cryopreserved
NIES-1085	Japan, Shimane Matsue Koshi-ike Pond	1984	Cryopreserved
NIES-1094	Japan, Lake Shirakaba	1984	Cryopreserved
NIES-1099	Japan, Noborio-ike Pond	1988	Cryopreserved
NIES-1101	Nepal, Kathmandu	1988	Cryopreserved
NIES-1104	Japan,Lake Barato	1989	Cryopreserved
NIES-1105	Japan, Lake Barato	1989	Cryopreserved
NIES-1113	Japan, Lake Suwa	1989	Cryopreserved
NIES-1115	Japan, Showa-tameike Pond	1989	Cryopreserved
NIES-1126	Japan, Lake Ohnuma	1989	Cryopreserved
NIES-1134	Japan, Lake Teganuma	1989	Cryopreserved
NIES-1135	Japan, Lake Teganuma	1989	Cryopreserved
NIES-1144	Japan, Lake Okutama (1989-09-17)	1989	Cryopreserved
NIES-1151	Japan, Nagano Chino Lake Shirakaba	1989	Cryopreserved
NIES-1157	Japan, Lake Teganuma	1989	Cryopreserved

NIES-1170	Japan, Lake Barato	1990	Cryopreserved
NIES-1211	Japan Hokkaido Abashiri Lake Tofutsu	1990	-
NIES-1213	Japan Okinawa Yonaguni Kunma Dam	1990	Cryopreserved
NIES-1215	Japan Okinawa Yonaguni Kunma Dam	1990	Cryopreserved
NIES-1216	Japan Okinawa Yonaguni Kunma Dam	1990	Cryopreserved
NIES-1217	Japan Okinawa Yonaguni Kunma Dam	1990	Cryopreserved
NIES-1218	Japan, Kunma Dam	1990	Cryopreserved
NIES-1222	Japan, Tatsugami Dam	1990	Cryopreserved
NIES-1224	Japan, Tatsugami Dam	1990	Cryopreserved
NIES-1230	Japan Okinawa Ishigaki Ishigaki Dam	1990	Cryopreserved
NIES-1232	Japan, Ishigaki Dam	1990	Cryopreserved
NIES-1233	Japan, Ishigaki Dam	1990	Cryopreserved
NIES-1239	Japan Okinawa Ishikawa Tengan Dam	1990	Cryopreserved

Toxicity (Microcysti	*mcy* G
-	-
-	-
+	+
+	+
+	+
-	-
-	-
-	-
-	-
+	+
-	-
-	-
-	-
+	+
-	-
+	+
-	-
-	-
+	+
-	-
+	+
-	-
-	-
+	+
+	+
+	+
-	-
-	-
+	+
-	-
+	+
-	-
-	-
+	+
-	+
-	-
+	+
-	+
-	+
-	-
+	+
-	+

-	-
-	-
+	+
+	+
-	-
-	-
-	-
+	+
-	-
+	+
-	-
-	-
-	-
