# Supplementary material for: Rapid Classification and Identification of Microcystis aeruginosa Strains Using MALDI–TOF MS and Polygenetic Analysis
Source: PLoS One. 2016 May 26;11(5):e0156275. doi: 10.1371/journal.pone.0156275 (PMC4881969; doi:10.1371/journal.pone.0156275)
Supplement: S2 Table — (PDF) [file pone.0156275.s002.pdf]

Table S 2 Ribosomal protein peak typing of 55 strains

| key | L32 | S21 | L33 | L35 | S18 | L29 | L31 |
|---|---|---|---|---|---|---|---|
| NIES-843 A | 1 | 1 | 1 | 1 | 1 | 1 | 1 |
| NIES-88 ( A | 1 | 0 | 0 | 0 | 1 | 6 | 0 |
| NIES-108 A | 1 | 1 | 0 | 0 | 1 | 6 | 1 |
| NIES-89 ( A | 1 | 1 | 1 | 1 | 1 | 1 | 1 |
| NIES-107 A | 1 | 1 | 1 | 1 | 1 | 1 | 1 |
| NIES-105 A | 1 | 1 | 1 | 1 | 1 | 1 | 1 |
| NIES-90 ( A | 1 | 1 | 1 | 1 | 1 | 1 | 1 |
| NIES-123 B | 2 | 1 | 1 | 1 | 1 | 2 | 2 |
| NIES-121 B | 1 | 1 | 1 | 2 | 1 | 2 | 2 |
| NIES-121 B | 2 | 1 | 1 | 2 | 1 | 2 | 2 |
| NIES-106 B | 1 | 1 | 1 | 2 | 1 | 2 | 2 |
| NIES-107 B | 1 | 1 | 1 | 2 | 1 | 2 | 2 |
| NIES-122 B | 2 | 1 | 0 | 2 | 1 | 2 | 2 |
| NIES-113 B | 1 | 1 | 1 | 2 | 0 | 2 | 2 |
| NIES-113 B | 1 | 1 | 1 | 2 | 0 | 2 | 2 |
| NIES-115 B | 1 | 1 | 1 | 2 | 0 | 2 | 2 |
| NIES-111 B | 1 | 1 | 1 | 2 | 1 | 2 | 2 |
| NIES-110 B | 2 | 1 | 0 | 2 | 1 | 2 | 2 |
| NIES-478 B | 2 | 1 | 1 | 3 | 1 | 2 | 2 |
| NIES-112 B | 0 | 1 | 0 | 0 | 1 | 2 | 2 |
| NIES-107 B | 2 | 1 | 1 | 2 | 1 | 7 | 2 |
| NIES-101 B | 2 | 1 | 1 | 2 | 1 | 0 | 2 |
| NIES-106 B | 2 | 1 | 0 | 1 | 1 | 0 | 2 |
| NIES-111 B | 1 | 1 | 1 | 2 | 1 | 7 | 2 |
| NIES-110 B | 2 | 1 | 0 | 2 | 1 | 2 | 1 |
| NIES-117 C | 1 | 1 | 0 | 0 | 1 | 4 | 0 |
| NIES-109 C | 0 | 1 | 1 | 1 | 1 | 4 | 0 |
| NIES-604 C | 0 | 1 | 0 | 0 | 1 | 2 | 0 |
| NIES-104 C | 0 | 1 | 0 | 2 | 1 | 7 | 0 |
| NIES-110 C | 1 | 1 | 0 | 0 | 1 | 4 | 0 |
| NIES-107 D | 0 | 1 | 0 | 2 | 1 | 6 | 0 |
| NIES-121 D | 0 | 1 | 1 | 0 | 0 | 6 | 1 |
| NIES-121 D | 0 | 0 | 1 | 0 | 0 | 6 | 1 |
| NIES-121 D | 0 | 1 | 0 | 0 | 0 | 0 | 0 |
| NIES-122 D | 0 | 0 | 0 | 2 | 0 | 7 | 0 |
| NIES-123 D | 0 | 0 | 0 | 0 | 1 | 6 | 1 |
| NIES-123 D | 0 | 0 | 0 | 0 | 1 | 7 | 1 |
| NIES-105 D | 0 | 1 | 1 | 2 | 1 | 6 | 0 |
| NIES-123 D | 0 | 1 | 1 | 2 | 1 | 6 | 1 |
| NIES-901 D | 0 | 1 | 0 | 0 | 1 | 6 | 1 |
| NIES-106 D | 0 | 1 | 1 | 3 | 0 | 6 | 1 |
| NIES-100 D | 1 | 1 | 1 | 0 | 0 | 6 | 1 |
| NIES-109 D | 0 | 1 | 1 | 0 | 0 | 6 | 1 |

| | | | | | | | |
|---|---|---|---|---|---|---|---|
| NIES-111 E | 1 | 1 | 1 | 0 | 1 | 6 | 1 |
| NIES-44 ( E | 1 | 1 | 1 | 0 | 1 | 6 | 1 |
| NIES-115 E | 1 | 1 | 0 | 0 | 1 | 2 | 1 |
| NIES-114 E | 0 | 0 | 0 | 0 | 0 | 0 | 3 |
| NIES-105 E | 1 | 0 | 0 | 0 | 0 | 0 | 3 |
| NIES-904 E | 0 | 0 | 0 | 0 | 0 | 0 | 3 |
| NIES-112 E | 0 | 0 | 1 | 0 | 0 | 0 | 3 |
| NIES-104 N | 1 | 1 | 0 | 1 | 1 | 0 | 1 |
| NIES-107 N | 1 | 1 | 1 | 0 | 1 | 1 | 1 |
| NIES-109 N | 2 | 1 | 1 | 0 | 1 | 2 | 2 |
| NIES-87 ( O | 1 | 1 | 1 | 0 | 1 | 2 | 3 |
| NIES-121 O | 1 | 1 | 0 | 0 | 1 | 0 | 2 |
| PCC 7941-t | 1 | 1 | 1 | 2 | 1 | 2 | 2 |
| PCC 9432-t | 0 | 1 | 1 | 3 | 1 | 2 | 2 |
| PCC 9443-t | 0 | 1 | 1 | 3 | 1 | 3 | 2 |
| PCC 9701-t | 2 | 1 | 1 | 1 | 1 | 4 | 3 |
| PCC 9806-n | 1 | 1 | 1 | 1 | 1 | 3 | 1 |

| L28 | S17 | S16 | S19 | S15 | S20 | S14 | L24 | L17 |
|---|---|---|---|---|---|---|---|---|
| 1 | 1 | 1 | 1 | 1 | 1 | 1 | 1 | 1 |
| 1 | 0 | 1 | 1 | 1 | 1 | 1 | 0 | 4 |
| 1 | 0 | 1 | 1 | 1 | 1 | 2 | 0 | 1 |
| 1 | 0 | 1 | 1 | 1 | 1 | 1 | 0 | 1 |
| 1 | 0 | 0 | 0 | 1 | 4 | 1 | 2 | 3 |
| 1 | 0 | 1 | 1 | 1 | 1 | 1 | 2 | 4 |
| 1 | 0 | 0 | 0 | 1 | 4 | 1 | 2 | 0 |
| 1 | 1 | 1 | 1 | 2 | 1 | 2 | 2 | 0 |
| 1 | 1 | 1 | 1 | 2 | 1 | 2 | 2 | 0 |
| 1 | 0 | 1 | 1 | 2 | 1 | 2 | 3 | 0 |
| 1 | 1 | 1 | 1 | 2 | 1 | 2 | 2 | 0 |
| 1 | 1 | 1 | 1 | 2 | 1 | 2 | 2 | 0 |
| 1 | 1 | 1 | 1 | 2 | 1 | 2 | 2 | 0 |
| 1 | 1 | 1 | 1 | 3 | 1 | 2 | 2 | 1 |
| 1 | 1 | 1 | 1 | 3 | 1 | 2 | 2 | 1 |
| 1 | 1 | 1 | 1 | 3 | 1 | 2 | 2 | 1 |
| 2 | 1 | 1 | 1 | 3 | 1 | 2 | 2 | 1 |
| 1 | 1 | 1 | 0 | 1 | 1 | 2 | 2 | 1 |
| 2 | 1 | 0 | 1 | 2 | 5 | 2 | 2 | 1 |
| 1 | 1 | 1 | 1 | 2 | 2 | 1 | 2 | 3 |
| 1 | 1 | 1 | 1 | 2 | 2 | 2 | 3 | 1 |
| 1 | 1 | 1 | 0 | 1 | 5 | 2 | 3 | 1 |
| 2 | 1 | 1 | 0 | 1 | 5 | 1 | 3 | 0 |
| 1 | 1 | 1 | 0 | 1 | 2 | 2 | 3 | 1 |
| 0 | 0 | 0 | 1 | 2 | 3 | 1 | 2 | 3 |
| 2 | 1 | 1 | 1 | 2 | 2 | 0 | 1 | 4 |
| 2 | 1 | 1 | 1 | 2 | 4 | 0 | 0 | 3 |
| 2 | 0 | 1 | 1 | 2 | 4 | 1 | 0 | 3 |
| 2 | 0 | 1 | 1 | 2 | 4 | 2 | 0 | 0 |
| 2 | 1 | 1 | 1 | 1 | 4 | 0 | 0 | 2 |
| 1 | 0 | 0 | 1 | 1 | 1 | 1 | 0 | 0 |
| 1 | 1 | 0 | 1 | 1 | 5 | 2 | 0 | 0 |
| 0 | 0 | 0 | 1 | 1 | 5 | 0 | 0 | 0 |
| 0 | 0 | 0 | 1 | 1 | 5 | 0 | 0 | 0 |
| 0 | 0 | 0 | 1 | 1 | 5 | 0 | 0 | 0 |
| 0 | 0 | 0 | 1 | 1 | 5 | 0 | 0 | 0 |
| 0 | 0 | 0 | 1 | 1 | 5 | 0 | 0 | 0 |
| 1 | 1 | 0 | 1 | 1 | 5 | 2 | 0 | 0 |
| 1 | 1 | 0 | 1 | 1 | 5 | 2 | 0 | 0 |
| 1 | 1 | 0 | 1 | 1 | 5 | 1 | 0 | 0 |
| 1 | 0 | 0 | 1 | 1 | 5 | 2 | 0 | 2 |
| 0 | 0 | 0 | 1 | 1 | 5 | 0 | 0 | 0 |
| 0 | 0 | 1 | 1 | 2 | 5 | 0 | 0 | 0 |

| | | | | | | | | |
|---|---|---|---|---|---|---|---|---|
| 1 | 0 | 1 | 1 | 1 | 1 | 0 | 3 | 3 |
| 1 | 0 | 1 | 1 | 1 | 1 | 0 | 0 | 3 |
| 1 | 1 | 1 | 1 | 1 | 1 | 2 | 0 | 1 |
| 1 | 0 | 0 | 0 | 2 | 2 | 0 | 0 | 0 |
| 0 | 0 | 0 | 0 | 2 | 2 | 0 | 0 | 0 |
| 3 | 0 | 0 | 0 | 2 | 2 | 0 | 0 | 0 |
| 0 | 0 | 0 | 0 | 2 | 2 | 0 | 0 | 0 |
| 1 | 0 | 1 | 1 | 1 | 1 | 1 | 2 | 0 |
| 1 | 0 | 1 | 1 | 1 | 1 | 1 | 0 | 1 |
| 1 | 1 | 1 | 1 | 2 | 2 | 1 | 3 | 1 |
| 2 | 1 | 0 | 1 | 1 | 1 | 2 | 3 | 3 |
| 1 | 1 | 0 | 1 | 1 | 1 | 2 | 3 | 3 |
| 2 | 1 | 1 | 1 | 2 | 1 | 2 | 2 | 1 |
| 1 | 1 | 1 | 1 | 2 | 2 | 2 | 2 | 2 |
| 3 | 1 | 1 | 1 | 2 | 3 | 2 | 3 | 3 |
| 1 | 2 | 2 | 1 | 1 | 4 | 2 | 4 | 4 |
| 1 | 1 | 2 | 1 | 3 | 5 | 2 | 3 | 1 |

| L22 | L18 | L14 | L19 | S12 | S13 |
|---|---|---|---|---|---|
| 1 | 1 | 1 | 1 | 1 | 1 |
| 1 | 1 | 1 | 1 | 1 | 1 |
| 2 | 0 | 1 | 1 | 1 | 1 |
| 1 | 1 | 1 | 1 | 1 | 1 |
| 2 | 1 | 1 | 0 | 1 | 1 |
| 1 | 1 | 1 | 1 | 1 | 1 |
| 2 | 4 | 1 | 0 | 1 | 1 |
| 2 | 1 | 1 | 1 | 1 | 1 |
| 2 | 1 | 1 | 2 | 1 | 0 |
| 2 | 1 | 1 | 3 | 1 | 1 |
| 2 | 1 | 1 | 3 | 1 | 1 |
| 2 | 2 | 1 | 1 | 1 | 1 |
| 1 | 0 | 1 | 3 | 1 | 1 |
| 2 | 1 | 1 | 1 | 1 | 1 |
| 2 | 1 | 1 | 1 | 1 | 1 |
| 2 | 1 | 1 | 1 | 1 | 1 |
| 2 | 1 | 1 | 2 | 1 | 1 |
| 2 | 1 | 1 | 2 | 1 | 1 |
| 2 | 1 | 1 | 2 | 1 | 1 |
| 1 | 0 | 1 | 2 | 0 | 1 |
| 2 | 1 | 1 | 2 | 1 | 1 |
| 2 | 2 | 1 | 2 | 1 | 1 |
| 2 | 2 | 0 | 0 | 1 | 1 |
| 2 | 2 | 1 | 2 | 1 | 1 |
| 2 | 4 | 0 | 1 | 1 | 1 |
| 2 | 0 | 1 | 1 | 1 | 1 |
| 2 | 0 | 1 | 1 | 1 | 1 |
| 1 | 0 | 1 | 1 | 1 | 1 |
| 1 | 1 | 1 | 1 | 1 | 1 |
| 2 | 0 | 0 | 0 | 1 | 1 |
| 2 | 0 | 1 | 0 | 0 | 1 |
| 1 | 0 | 1 | 0 | 1 | 1 |
| 1 | 0 | 1 | 0 | 0 | 0 |
| 0 | 0 | 1 | 0 | 1 | 1 |
| 0 | 0 | 1 | 0 | 0 | 0 |
| 0 | 0 | 1 | 0 | 1 | 1 |
| 0 | 0 | 1 | 0 | 0 | 1 |
| 0 | 3 | 1 | 0 | 1 | 0 |
| 0 | 3 | 1 | 0 | 1 | 0 |
| 0 | 3 | 1 | 0 | 1 | 0 |
| 1 | 0 | 0 | 2 | 1 | 0 |
| 1 | 0 | 1 | 0 | 0 | 0 |
| 1 | 0 | 1 | 2 | 0 | 0 |

| | | | | | |
|---|---|---|---|---|---|
| 2 | 1 | 1 | 1 | 1 | 1 |
| 2 | 1 | 1 | 1 | 1 | 1 |
| 2 | 4 | 1 | 1 | 1 | 1 |
| 1 | 0 | 0 | 2 | 0 | 0 |
| 1 | 0 | 0 | 2 | 0 | 0 |
| 1 | 0 | 0 | 2 | 0 | 0 |
| 1 | 0 | 0 | 2 | 0 | 0 |
| 1 | 1 | 0 | 1 | 1 | 1 |
| 1 | 1 | 1 | 1 | 1 | 1 |
| 2 | 0 | 1 | 2 | 0 | 1 |
| 2 | 0 | 1 | 2 | 0 | 1 |
| 0 | 2 | 0 | 0 | 1 | 1 |
| 2 | 2 | 1 | 2 | 1 | 1 |
| 2 | 1 | 1 | 2 | 1 | 1 |
| 2 | 3 | 1 | 1 | 1 | 1 |
| 2 | 1 | 1 | 1 | 1 | 1 |
| 2 | 4 | 1 | 3 | 1 | 1 |
